# Nrf2, the Major Regulator of the Cellular Oxidative Stress Response, is Partially Disordered

**DOI:** 10.3390/ijms22147434

**Published:** 2021-07-11

**Authors:** Nadun C. Karunatilleke, Courtney S. Fast, Vy Ngo, Anne Brickenden, Martin L. Duennwald, Lars Konermann, Wing-Yiu Choy

**Affiliations:** 1 Department of Biochemistry, The University of Western Ontario, London, ON N6A 5C1, Canada; nkarunat@uwo.ca (N.C.K.); abricken@uwo.ca (A.B.); 2 Department of Chemistry, The University of Western Ontario, London, ON N6A 5B7, Canada; courtfast@gmail.com; 3 Department of Pathology and Laboratory Medicine, The University of Western Ontario, London, ON N6A 5C1, Canada; vngo23@uwo.ca (V.N.); martin.duennwald@schulich.uwo.ca (M.L.D.)

**Keywords:** oxidative stress, Nrf2, Keap1, nuclear magnetic resonance spectroscopy, hydrogen/deuterium exchange, mass spectrometry, circular dichroism, intrinsically disordered

## Abstract

Nuclear factor erythroid 2-related factor 2 (Nrf2) is a transcription regulator that plays a pivotal role in coordinating the cellular response to oxidative stress. Through interactions with other proteins, such as Kelch-like ECH-associated protein 1 (Keap1), CREB-binding protein (CBP), and retinoid X receptor alpha (RXRα), Nrf2 mediates the transcription of cytoprotective genes critical for removing toxicants and preventing DNA damage, thereby playing a significant role in chemoprevention. Dysregulation of Nrf2 is linked to tumorigenesis and chemoresistance, making Nrf2 a promising target for anticancer therapeutics. However, despite the physiological importance of Nrf2, the molecular details of this protein and its interactions with most of its targets remain unknown, hindering the rational design of Nrf2-targeted therapeutics. With this in mind, we used a combined bioinformatics and experimental approach to characterize the structure of full-length Nrf2 and its interaction with Keap1. Our results show that Nrf2 is partially disordered, with transiently structured elements in its Neh2, Neh7, and Neh1 domains. Moreover, interaction with the Kelch domain of Keap1 leads to protection of the binding motifs in the Neh2 domain of Nrf2, while the rest of the protein remains highly dynamic. This work represents the first detailed structural characterization of full-length Nrf2 and provides valuable insights into the molecular basis of Nrf2 activity modulation in oxidative stress response.

## 1. Introduction

Reactive oxygen species (ROS) from the environment or generated by the cellular metabolism can cause oxidative damage to proteins, DNA, and lipids, leading to diseases such as cancer, dementia, and cardiovascular disease, to name a few [1,2]. Nuclear factor erythroid 2-related factor 2 (Nrf2) is an essential transcription factor for protecting cells from these harmful effects [3,4,5,6]. Through binding to the antioxidant-responsive element (ARE) in their promoter regions, Nrf2 induces the expression of numerous cytoprotective genes and safeguards cells from tumorigenesis [4,7]. On the other hand, aberrant activation of Nrf2 is associated with poor prognosis and chemoresistance of many cancer types [8,9,10,11,12,13,14]. Genomic characterization of squamous cell lung cancers revealed that the Nrf2 antioxidant pathway is one of the most severely altered pathways [15]. Many mutations of Nrf2 are expected to affect its target recognition [16,17], resulting in its dysregulation [18,19]. Thus, pharmacological modulation of Nrf2 activity represents an attractive strategy for cancer treatment [20].

Nrf2 activity is regulated through the interactions with a suite of different proteins [4,21,22,23,24,25,26,27], including Kelch-like ECH-associated protein 1 (Keap1) [22,28]. The 70 kDa Keap1, which acts as the primary negative regulator of Nrf2, consists of three major functional domains: the N-terminal BTB domain, the IVR region, and the C-terminal Kelch domain. Under homeostatic conditions, dimeric Keap1 binds Nrf2 via the Kelch domains and recruits it to the Cullin-3 based E3 ligase complex for ubiquitination, leading to the proteasomal degradation of Nrf2 [22,29,30]. In the presence of oxidative stress, several redox-sensitive cysteines in Keap1 (e.g., C151, C273, and C288) are modified by ROS, resulting in protein conformational changes and disruption of Nrf2 binding [31,32]. This leads to the accumulation of Nrf2 in the nucleus and subsequent activation of ARE-dependent gene transcription.

Nrf2 is a member of the Cap ‘n’ Collar (CNC) family of basic leucine-zipper transcription factors. The 68 kDa human Nrf2 comprises seven Nrf2-ECH homology (Neh) functional domains, known as Neh1–7 [25,33]. Note that for historical reasons, the domain numbers are not ordered according to the sequence [34] (Figure S2A). The N-terminal Neh2 domain binds the Kelch domains of dimeric Keap1 via a high-affinity ETGE motif and a low-affinity DLG motif [22,35]. Following the Neh2 domain in the protein sequence, Neh4 and Neh5 are the transactivation domains that recognize the transcription co-activator CBP [23,36], whereas the Neh7 domain binds to the negative regulator RXRα [25,37]. The Neh6 domain interacts with the β-transducin repeat-containing protein 1, which leads to Keap1-independent ubiquitination and degradation of Nrf2 [38,39]. The Neh1 domain mediates heterodimerization with sMaf proteins for DNA-binding [4,40], whereas the C-terminal Neh3 region is another transactivation domain associated with chromo ATPase/helicase DNA-binding protein 6 [41].

Despite its critical role in the antioxidant response, little is known about the molecular structure of Nrf2. To date, only the structures of the isolated N-terminal Neh2 domain (residues 1–98) and part of the Neh1 DNA-binding domain (residues 445–523; PDB: 2LZ1) have been experimentally characterized [22,28,42,43]. Nuclear magnetic resonance (NMR) studies revealed that the Neh2 domain is intrinsically disordered (i.e., it does not adopt a stable folded conformation) yet possesses transient local structural elements. In particular, the region of residues 39–71 that links the two Keap1-binding motifs (i.e., the DLG and ETGE elements) displays significant helical propensity [22].

The Neh1 DNA-binding domain comprises three regions: the CNC homology region, the basic DNA recognition motif, and the leucine-zipper region [33]. The solution structure of a 79-residue fragment (PDB: 2LZ1; residues 445–523 of human Nrf2) representing only part of the Neh1 domain (lacking the C-terminal leucine-zipper dimerization region) was solved using NMR spectroscopy [43]. The structure contains 4 α-helices (H1–H4; residues 455–465, 469–475, 478–489, and 491–505), whereas the remaining 34 residues (~43% of the structure) are disordered.

Gaining a mechanistic understanding of how Nrf2 is regulated through interactions with different binding partners requires a multi-pronged approach. In this work, we combined bioinformatics tools with various biophysical techniques, including hydrogen/deuterium exchange mass spectrometry (HDX-MS), circular dichroism (CD) spectropolarimetry, and NMR spectroscopy to investigate the structural properties of full-length Nrf2 (from now on referred to as FL-Nrf2). This is in contrast to earlier studies that were limited to truncated constructs [22,42,43]. Further, HDX-MS was used to characterize the interactions of Nrf2 with the Kelch domain of Keap1 (from now on referred to as “Kelch”). Intriguingly, our results reveal that FL-Nrf2 is partially disordered yet possesses several transiently structured elements. Upon binding with Kelch, the DLG and ETGE binding motifs in the Neh2 domain of Nrf2 became protected, yet the rest of the protein remained highly dynamic. These unique structural properties may be involved in regulating the interactions of Nrf2 with other proteins and thus determine its function in response to oxidative stress.

## 2. Results

### 2.1. Full-Length Nrf2 (FL-Nrf2) is Predicted to Be Partially Disordered

FL-Nrf2 is an acidic protein with pI ~4.8. At physiological pH, it is highly charged (~ –40 at pH 7). Using the optimized expression and purification protocols outlined under Materials and Methods, we were able to produce ~0.3 mg of recombinant full-length protein with high purity from 1 L of M9 culture. Intriguingly, although the molecular weight of FL-Nrf2 is only 70.4 kDa (including the His-tag), it runs with an apparent MW of ~110 kDa on SDS-PAGE gels as already reported previously [44] (Figure S1). The high net charge and aberrant SDS-PAGE migration behavior suggest that FL-Nrf2 may be partially disordered [45,46].

We used bioinformatics tools to further investigate the potential disorder of FL-Nrf2 (Figure S2). PONDR-FIT [47], a meta-protein disorder predictor that combines the results of six different methods, indicates that many regions of FL-Nrf2 are disordered (i.e., disorder disposition > 0.5, Figure S2A). In particular, the N-terminal Neh2 domain is predicted to be largely unstructured, in agreement with published data [22]. Meanwhile, local structured elements are expected to be present in the Neh4 (112–134) and Neh5 domains (183–201), the two transactivation domains that bind CBP [23,36]. Neh6 (338–388) and Neh3 (562–605) are predicted to be mainly unstructured, whereas Neh7 (209–316) and Neh1 (435–562) appear to be partially disordered.

We also applied s2D, another sequence-based disorder predictor, to further examine the conformational propensities of FL-Nrf2. s2D predicts not only disordered regions but also estimates the secondary structure at the residue level [48]. The method predicted that ~90% of the residues in FL-Nrf2 predominantly adopt a random coil conformation (Figure S2B). While no residues were identified to sample the β-strand conformations, 57 residues (20–23, 479–486, 492–502, and 533–566) were predicted to have a helical propensity of >46%. Notably, the longest stretch (residues 533–566) that showed a preferentially helical conformation is in the Neh1 domain. DisEMBL [49] and IUPread2A [50], two other sequence-based structural predictors, were also used to characterize FL-Nrf2, all of which agree with the PONDR-FIT and s2D data (Figure S2C).

### 2.2. CD and NMR Experiments Confirm that FL-Nrf2 is Intrinsically Disordered

**Figure 1:**
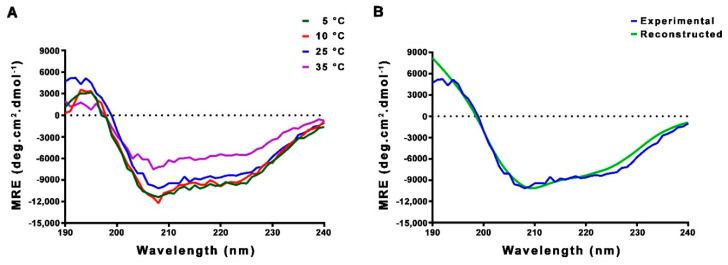
(**A**) CD spectra of FL-Nrf2 recorded at 5, 10, 25, and 35 °C. (**B**) Deconvolution of FL-Nrf2 CD spectrum at 25 °C using the CONTINLL program in DichroWeb, with protein reference data set 4 (optimized for 190–240 nm). The NRMSD between the experimental and reconstructed CD data is 0.13.

We next used CD and NMR techniques to validate our bioinformatics findings. CD spectropolarimetry was applied for assessing the secondary structure of FL-Nrf2 at 5, 10, 25, and 35 °C. The negative bands at 208 and 222 nm indicate the presence of α-helical structural elements (Figure 1A). Spectral deconvolution indicates that at 25 °C, there are around 27% α-helical, 21% β-strand and 52% disordered/turn structures, implying that Nrf2 is indeed partially disordered (Figure 1B; Table S1). While lowering the temperature to 5 or 10 °C did not considerably alter the secondary structure content, the percentage of α-helical structure dropped to ~18% when the temperature was increased to 35 °C.

**Figure 2:**
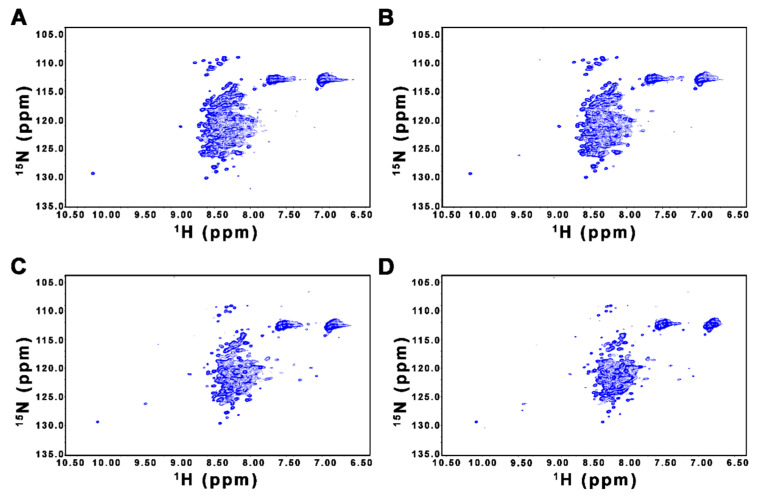
^1^H-^15^N HSQC spectra of FL-Nrf2 (20 μM) in 50 mM ammonium acetate buffer, 0.5 mM TCEP (pH 6.5) recorded at (**A**) 5 °C, (**B**) 10 °C, (**C**) 25 °C, and (**D**) 35 °C. Most of the observed backbone amide signals are crowded in a narrow region between 7.8 and 8.7 ppm in the ^1^H dimension at all four temperatures.

We used NMR spectroscopy to further verify the disordered nature of FL-Nrf2. Figure 2 shows the ^1^H-^15^N HSQC spectra of FL-Nrf2 acquired at 5, 10, 25, and 35 °C. At all four temperatures, despite the presence of some well-dispersed peaks with relatively weak intensities, most of the observed backbone amide signals are crowded in a narrow region between 7.8 and 8.7 ppm in the ^1^H dimension. This lack of ^1^H resonance dispersion indicates that many parts of FL-Nrf2 do not adopt stable structures and undergo rapid conformational interconversion [51,52,53,54]. In addition, the disordered nature of FL-Nrf2 does not change significantly at lower temperatures. For comparison, we also acquired the HSQC spectrum of FL-Nrf2 at 35 °C in the presence of 6 M urea, under which conditions the protein was expected to be largely unfolded (Figure S3). The similarity of all these spectra therefore suggest that FL-Nrf2 is already extensively unfolded even in the absence of urea. Although the signal overlap hampers further site-specific structural analyses of FL-Nrf2 by NMR spectroscopy, our CD and NMR results nevertheless demonstrate that FL-Nrf2 is significantly disordered.

**Figure 3:**
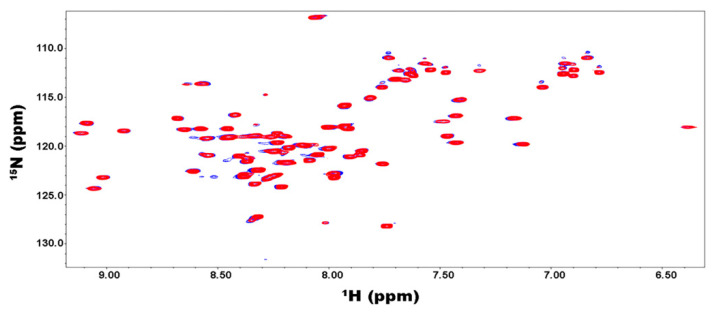
^1^H-^15^N HSQC NMR spectra of Neh1–2LZ1 (100 μM) in the presence (red) and absence (blue) of 50 mM arginine in 50 mM ammonium acetate buffer, 1 mM DTT, pH 6.5. The spectra were recorded at 25 °C.

The solution NMR structure of a 79-residue fragment (residues 445–523 of human Nrf2; denoted as Neh1–2LZ1; PDB accession: 2LZ1) representing part of the Neh1 domain shows that this segment is partially folded in isolation. Therefore, some well-dispersed peaks in the ^1^H-^15^N HSQC spectrum of FL-Nrf2 could originate from the Neh1–2LZ1 region. To test this possibility, we performed NMR and CD analyses on isolated Neh1–2LZ1. Figure 3 shows the ^1^H-^15^N HSQC NMR spectrum of Neh1–2LZ1 in 50 mM ammonium acetate (pH 6.5) with 50 mM arginine, the same condition used for solving the Neh1–2LZ1 structure. It has been shown that arginine can increase the stability and solubility of some proteins [55,56]. For completeness, we also acquired HSQC data of Neh1–2LZ1 in the absence of arginine (Figure 3). Both spectra contain well-dispersed peaks and are very similar, suggesting that isolated Neh1–2LZ1 is, to some extent, structured both in the presence and absence of arginine.

The CD spectrum of Neh1–2LZ1 depicts two negative minima at 208 and 222 nm, as well as a positive band at 195 nm (Figure S4), illustrating the existence of helical content. Deconvolution analysis estimated the population of α-helical, β-strand, and disordered/turn structures to be 39%, 14%, and 47%, respectively, consistent with the partially disordered nature of Neh1–2LZ1.

### 2.3. HDX-MS Reveals that Many Regions of FL-Nrf2 are Highly Dynamic

HDX-MS experiments were performed to further probe the conformational dynamics of FL-Nrf2 in a spatially resolved manner. Backbone amide hydrogen/deuterium exchange (HDX) coupled with ESI-MS is a powerful method for studying protein behavior in solution [57,58]. Regions that are involved in hydrogen-bonding networks or occluded from the solvent will undergo slow exchange. In contrast, regions that are disordered and solvent-accessible will undergo fast exchange upon protein exposure to D_2_O. The exchange process is due to dynamic fluctuations that disrupt backbone amide hydrogen bonds. By measuring deuterium incorporation, it is possible to determine the relative flexibility of backbone segments of a protein [59].

Pepsin digestion of FL-Nrf2 yielded 101 peptides resulting in 86.3% sequence coverage (Figure S5B). Fast HDX kinetics were observed throughout the entire protein, where the majority of the peptides exhibit ~100% deuteration after 12 s. This lack of protection indicates that FL-Nrf2 is significantly disordered (Figure S6). Notably, several peptides in distinct parts of the protein showed somewhat slower deuteration. For instance, the peptide covering residues 54–74 in the Neh2 domain only displayed complete deuteration after >24 s. This observation is consistent with earlier findings that even though the Neh2 domain is disordered, helical propensity exists between residues 39 and 71 [22]. Other regions that showed somewhat slower exchange are 235–249, 417–434, and 512–537. They are located in the Neh7 domain, the linker between the Neh6 and Neh1 domains, and the Neh1 domain, respectively.

Interestingly, the regions corresponding to three of the four helices (H1-H3; residues 455–465, 469–475, and 478–489) in the Neh1–2LZ1 structure did not show higher protection, suggesting that these helical regions undergo extensive conformational fluctuations, which would facilitate deuterium uptake. Another possible explanation is that the Neh1–2LZ1 region is less stable in FL-Nrf2.

### 2.4. Binding of the Kelch Domain of Keap1 (“Kelch”) Triggers Conformational Changes Localized in the Neh2 Domain of Nrf2

**Figure 4:**
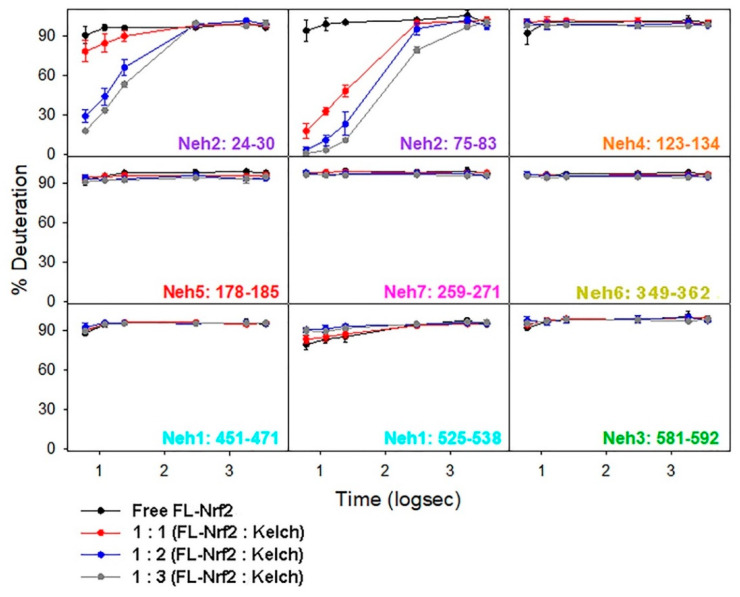
HDX-MS kinetic plots of free FL-Nrf2 (black) and in the presence of 1:1 (red). 1:2 (blue), and 1:3 (gray) molar ratios of Kelch.

We also investigated how FL-Nrf2 interacts with Kelch, which is part of the negative regulator Keap1. Previous studies showed that the isolated Neh2 domain of FL-Nrf2 binds Kelch at two sites: the high- affinity ETGE motif (around residues 76–84; K_d_ ~5 nM) and the low-affinity DLG motif (around residues 17–46; K_d_ ~1 μM) [22]. Here we used HDX-MS to further dissect the effects of Kelch-binding on the conformational dynamics of FL-Nrf2. In these experiments, the FL-Nrf2 concentration was held constant at 2 μM, while the Kelch concentration varied from 2 to 4 and 6 μM, which corresponded to FL-Nrf2:Kelch ratios of 1:1, 1:2, and 1:3, respectively (Figure 4). By testing these different concentration ratios, it should be possible to selectively saturate either only the high-affinity motif or both binding sites due to their distinct K_d_ values. Significant changes in deuterium uptake were observed in the Neh2 domain upon the addition of Kelch. In the presence of 1:1 Kelch, a considerable reduction in FL-Nrf2 deuterium uptake was displayed around the ETGE binding motif and a small reduction in deuterium uptake around the DLG motif, consistent with the binding affinities of the two sites. For a 1:2 ratio, a substantial reduction in deuterium uptake was observed around the DLG binding site and an even more significant reduction in HDX close to the ETGE motif. Further reduction in deuterium uptake around both sites was noted at a 1:3 ratio. Notably, the addition of Kelch did not slow down the deuterium uptake in any other domains of FL-Nrf2.

**Figure 5:**
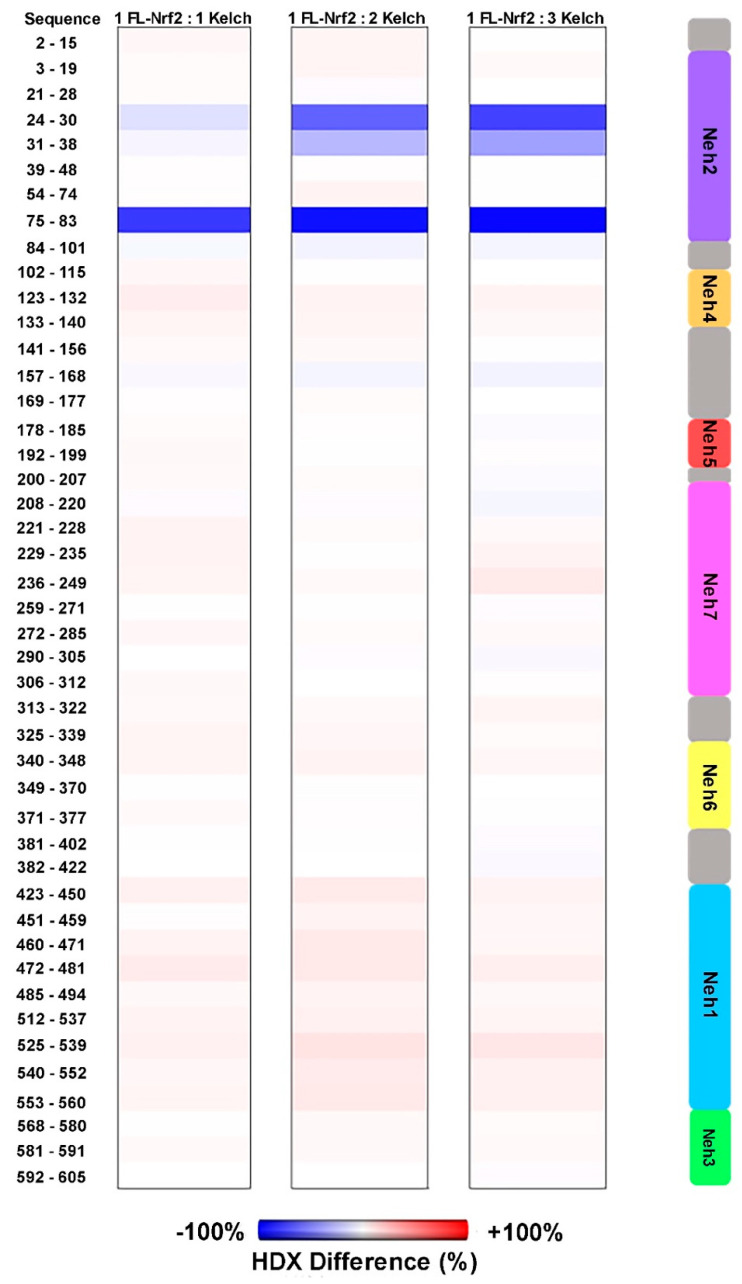
HDX-MS% difference plots of FL-Nrf2 upon addition of Kelch in 1:1, 1:2, and 1:3 ratios relative to free FL-Nrf2 at t = 6 s. Blue represents reduced deuteration, and red represents enhanced deuteration after the addition of Kelch. The side panel indicates the domain organization of FL-Nrf2.

Figure 5 shows % HDX differences at t = 6 s of FL-Nrf2 after addition of Kelch in 1:1, 1:2, and 1:3 ratios. In these maps, blue regions indicate less HDX than for free FL-Nrf2, whereas red regions indicate more HDX (more dynamic) than for free FL-Nrf2. These plots clearly display the enhanced protection of the Neh2 domain that was caused by increasing the Kelch concentration. Surprisingly, all the other Nrf2 domains experience slightly higher flexibility at the earliest time point upon binding the Kelch domain (red hues). These data suggest that when FL-Nrf2 binds to the Kelch domain, the rest of the protein becomes somewhat more dynamic. Our results, therefore, reveal that Kelch binds the Neh2 domain of FL-Nrf2 in a highly selective manner without triggering folding transitions in the rest of the protein.

### 2.5. Effects of Nrf2-Binding on the Deuterium Uptake of the Kelch Domain

**Figure 6:**
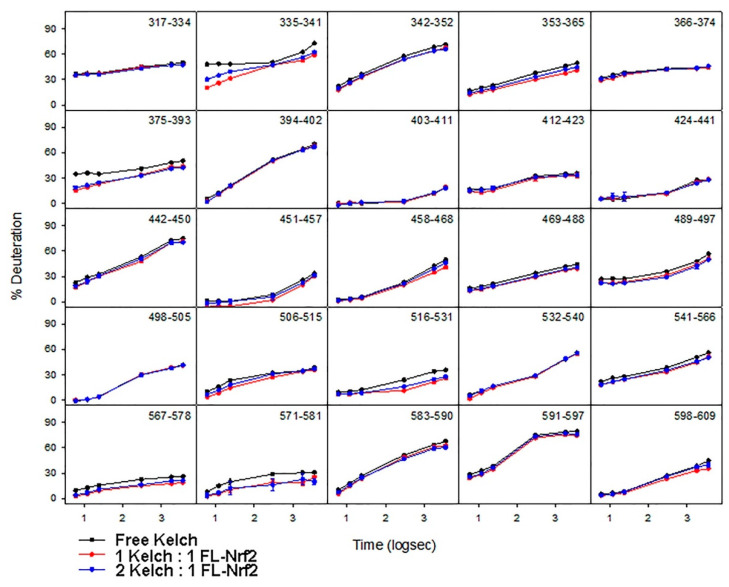
HDX-MS kinetic plots for free Kelch (black), 1:1 (red), and 2:1 (blue) Kelch:FL-Nrf2.

The deuteration kinetics of free Kelch (Figure 6; black) display slow uptake throughout its entire sequence, consistent with its folded β-propeller structure [28,60]. For 1:1 and 2:1 (Kelch: FL-Nrf2) conditions, the overall HDX kinetics remained the same throughout the entire Kelch sequence. Peptides 335–341, 375–393, and 571–581 are the only three regions that showed a slight reduction in deuterium uptake when FL-Nrf2 was added. Peptide 375–393 contains residue N382 that is known to form a hydrogen bond with the ETGE motif of the Neh2 domain of Nrf2 [28,60]. Our HDX data are consistent with this behavior, as peptide 375–393 displayed a reduction in deuterium uptake indicative of protection of this site.

**Figure 7:**
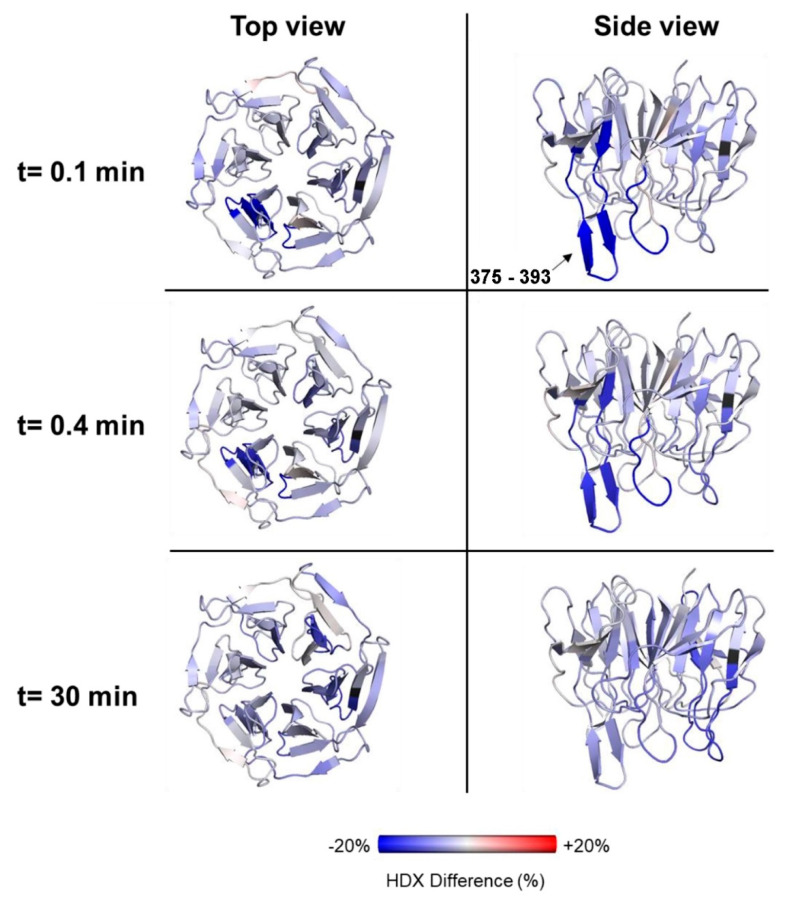
HDX % difference plots for Kelch upon addition of FL-Nrf2 at 0.1, 0.4, and 30 min compared to free Kelch. Blue represents reduced deuteration after the addition of FL-Nrf2.

To understand the effect of Nrf2-binding on the global dynamics of Kelch, the HDX difference of the 1:1 state was plotted at time points 0.1, 0.4, and 30 min compared to free Kelch (Figure 7). At 0.1 and 0.4 min, a large reduction in deuterium uptake was observed in peptides 375–393, consistent with hydrogen bond formation of this site when the Kelch domain interacts with FL-Nrf2. Overall, the data show that there is a general stabilization of the entire Kelch domain upon binding to FL-Nrf2.

## 3. Discussion

Nrf2 is a key transcription factor that orchestrates cellular responses to oxidative stress [3,6,61]. Aberrant activation of Nrf2 has been shown to play a pivotal role in pathogenesis and chemoresistance for many types of cancer [18,62]. Recent studies revealed that dysregulation of Nrf2 is also associated with neurodegenerative disorders and cardiovascular disease [63,64,65,66]. The involvement of Nrf2 in these human diseases makes the pharmacological modulation of Nrf2 activity a promising therapeutic strategy. Indeed, since the structures of Kelch in complex with the ETGE and DLG peptides derived from Nrf2 became available [28,42,60], tremendous efforts have been devoted to the design of small molecules and peptides that can inhibit the Nrf2-Kelch interaction with high specificity [26,67]. Unraveling the mechanism of Nrf2 binding to other regulators, such as CBP and RXRα, will no doubt open up additional opportunities for developing effective therapeutic strategies. Our work represents the first structural characterization of Nrf2 in the full-length context, which is a critical step toward this goal.

We have used various bioinformatics tools to predict the structural characteristics of FL-Nrf2. The results suggest that FL-Nrf2 is significantly disordered, although local structural elements exist in specific regions of the protein. Our bioinformatics findings are supported by extensive biophysical characterization using CD, NMR, and HDX-MS techniques. The CD results confirm that FL-Nrf2 is partially disordered but displays a significant helical propensity. The results are consistent with the lack of peak dispersion observed in the ^1^H-^15^N HSQC NMR spectrum of FL-Nrf2.

Our HDX-MS results provide a clearer picture of the FL-Nrf2 dynamics. With an ~86% overall sequence coverage, we were able to probe the conformational dynamics of different FL-Nrf2 domains. Even though the majority of the peptides produced by pepsin digestion showed fast deuterium uptake, a few regions were found to be moderately protected. Unexpectedly, peptides in the Neh1–2LZ1 region did not display high protection. It is possible that even though isolated Neh1–2LZ1 is partially structured, conformational fluctuations still allow for relatively fast deuterium uptake.

The data presented in this work highlight the importance of using full-length Nrf2 to uncover the structural and dynamic characteristics of this protein, as opposed to earlier studies that used fragments or protein truncations. By using the FL-Nrf2 construct, we uncovered important conformational properties that provide insights into the biological role of Nrf2. Our results show that free FL-Nrf2 is highly dynamic throughout its entire sequence. When FL-Nrf2 interacts with Kelch, a large reduction in deuterium uptake is observed in the DLG and ETGE binding motifs of FL-Nrf2. Other parts of FL-Nrf2 became slightly more dynamic, as indicated by an increase in HDX. We also found that the interaction of FL-Nrf2 with Kelch resulted in a stabilization of the entire Kelch domain.

The disordered nature of FL-Nrf2 has substantial implications for its biological function. Intrinsically disordered proteins are highly abundant in all organisms [68]. Like FL-Nrf2, many of these proteins are involved in gene transcription and signal transduction [69,70]. Distinct from globular proteins, disordered proteins do not adopt a well-defined structure under physiological conditions. Instead, they exist as a large population of conformations in dynamic equilibria that can shift upon changes in the environment [51,71]. The structural plasticity of FL-Nrf2 can confer functional advantages. For instance, the lack of a stable tertiary fold allows FL-Nrf2 to bind multiple targets using a number of linear motifs located in different protein regions, either simultaneously or sequentially, without steric restrictions [72,73]. This aligns with our HDX-MS results showing that the effects of Kelch-binding on the structure of FL-Nrf2 are localized to the Neh2 domain in a highly specific manner. Further, the conformational dynamics of FL-Nrf2 can also have substantial consequences for its target recognition. Upon complex formation, the unfavorable entropy loss due to the folding into more stable bound-state conformations of FL-Nrf2 must be offset by strong enthalpic interactions with the binding partner. This enthalpy-entropy compensation confers Nrf2 the ability to bind distinct targets with high specificity and low affinity, which is essential for its regulation through various protein-protein interactions [74,75,76].

## 4. Materials and Methods

### 4.1. Protein Expression and Purification of Full-Length Human Nrf2

The construct of full-length human Nrf2 (FL-Nrf2; purchased from Invitrogen^®^) cloned into the Gateway Destination Vector pDEST17 was transformed into *E. coli* (Rosetta 2(DE3) pLysS) cells for protein expression. The cell culture was incubated in M9 minimal media (47.8 mM of Na_2_HPO_4_, 22.0 mM of KH_2_PO_4_, 8.6 mM of NaCl, 0.1 mM of CaCl_2_, 2.0 mM of MgSO_4_, 10 mg of biotin, 10 mg of thiamin, 4.0 g of glucose, and 1.0 g of NH_4_Cl; pH 7.4) at 37 °C until the OD600 reached ~0.8. Protein over-expression was induced with 1 mM isopropyl-β-D-thiogalactopyranoside (IPTG). To avoid purifying Nrf2 from inclusion bodies through refolding procedures, we have tested four different expression temperatures (15, 22, 30, and 37 °C) and two induction times (5 and 18 h) to identify conditions that maximize the amount of FL-Nrf2 in the soluble fraction. Our data showed that induction at higher temperatures (i.e., 30 and 37 °C) for 5 or 18 h resulted in the majority of FL-Nrf2 in the insoluble fraction. In contrast, most of the protein was found in the soluble fraction when cells were induced at 15 °C for 18 h.

The cell pellets were resuspended using solubilization buffer (20 mM Tris-HCl, 150 mM NaCl, 1 mM EDTA, 5 mM 2-mercaptoethanol, pH 8.1). Lysozyme was added to the solubilized cell suspension, and the mixture was incubated for 30 min at 37 °C. The incubated sample was homogenized using an Avestin EmulsiFlex-C5 homogenizer. A SigmaFast Protease Inhibitor Cocktail tablet (EDTA-free) and 1 mM PMSF (100 μL per 10 mL of the lysed sample) were added to the sample. Final concentrations of imidazole (10 mM) and NaCl (500 mM) were adjusted, and the sample was centrifuged at 40,000× *g* for 30 min at 4 °C. The supernatant was collected, and the pH was adjusted to 7.4–7.8. The sample was loaded onto equilibrated Ni-Sepharose 6 fast flow beads (GE Healthcare) and incubated for 2 h at room temperature. The sample was then washed with 400 mL of primary wash buffer (20 mM Tris-HCl, 500 mM NaCl, 80 mM imidazole, 5 mM 2-mercaptoethanol, pH 7.8), followed by 10 mL (5 mL × 2) of secondary wash buffer (20 mM Tris-HCl, 500 mM NaCl, 150 mM imidazole, 5 mM 2-mercaptoethanol, pH 7.8). The protein was then eluted using 5-mL fractions of elution buffer (20 mM Tris-HCl, 500 mM NaCl, 1.5 M imidazole, 5 mM 2-mercaptoethanol, pH 7.8), and the eluate was monitored using Bradford assay (Bio-Rad, Hercules, CA, USA). Fractions containing FL-Nrf2 were pooled and dialyzed overnight into the dialysis buffer (50 mM ammonium acetate, 500 μM TCEP, pH 6.5). The final protein concentration was determined by the Lowry assay. By using this new protocol, we were able to obtain about 0.3 mg of purified FL-Nrf2 from a 1 L M9 minimal media culture. Notably, purified FL-Nrf2 does not have very high solubility. The solubility of FL-Nrf2 was analyzed by the sedimentation assay (the procedure of the assay is outlined in Supplemental Materials) to partition the soluble and aggregated protein molecules into supernatant and pellet for analysis. The results show that FL-Nrf2 in 50 mM ammonium acetate and 0.5 mM TCEP (pH 6.5) was only present in the supernatant but not in the pellet (Figure S7), confirming that the protein does not aggregate at concentrations <20 μM. Therefore, samples with a concentration of <20 µM were used in our studies.

### 4.2. Expression and Purification DNA-Binding Neh1 Domain of Nrf2 (Neh1–2LZ1, Residues 445–523)

The Neh1–2LZ1 construct was purchased from the Northeast Structural Genomics Consortium. It was cloned into the Gateway Destination Vector pDEST17 with a tobacco etch virus (TEV) protease cleaving site and transformed into BL21(DE3) for expression. The cell culture was incubated in M9 minimal media at 37 °C until the OD600 reached ~0.8. Protein over-expression was induced with 1 mM IPTG. Cells were then grown overnight at 17 °C before harvest by centrifugation.

The cell pellets were resuspended in denaturing Ni^2+^ binding buffer (25 mM Tris-HCl, 250 mM NaCl, 8 M urea, 5 mM 2-mercaptoethanol, pH 8.0). The cell suspension was homogenized by Dounce homogenization and sonication. The mixture was then centrifuged at 50,000× *g* at room temperature for 40 min. Ni-Sepharose 6 fast flow beads (GE Healthcare) pre-equilibrated with binding buffer were added to the supernatant, and the mixture was incubated for 2 h at room temperature. The mixture was loaded onto a column and washed with 200 mL of primary wash buffer (25 mM Tris-HCl, 250 mM NaCl, 10 mM imidazole, 8 M urea, 5 mM 2-mercaptoethanol, pH 8.0), followed by a wash with 200 mL of secondary wash buffer (25 mM Tris-HCl, 250 mM NaCl, 10 mM imidazole, 5 mM 2-mercaptoethanol, pH 8.0). The protein was eluted using 5 mL fractions of elution buffer (25 mM Tris-HCl, 500 mM NaCl, 750 mM imidazole, 5 mM 2-mercaptoethanol, pH 7.8), and eluted fractions were monitored using Bradford assay. Fractions containing Neh1–2LZ1 were pooled and dialyzed overnight into the HEPES buffer (20 mM HEPES, 5 mM 2-mercaptoethanol, pH 8.0) at 4 °C. The next day, the buffer was refreshed, and the sample was dialyzed for another 4 h before the protein concentration was determined using Bradford assay. TEV protease was then added accordingly (1 mg of TEV protease/25 mg of protein). Following overnight incubation at room temperature, the sample was diluted into the HEPES buffer and was loaded onto a pre-equilibrated SP-Sepharose (GE Healthcare) column and incubated at room temperature for one hour. After incubation, the sample was washed with 200 mL of the third wash buffer (20 mM HEPES, 50 mM NaCl, 5 mM 2-mercaptoethanol, pH 8.0). Finally, protein was eluted using the elution buffer (20 mM HEPES, 500 mM NaCl, 5 mM 2-mercaptoethanol, pH 8.0) in 5 mL fractions. Eluted fractions were pooled and dialyzed into the NMR buffer (50 mM ammonium acetate, 1 mM DTT, pH 6.5) in the presence or absence of 50 mM of arginine.

### 4.3. Expression and Purification Kelch Domain of Human Keap1

The pET15b plasmid of human Keap1-Kelch cDNA, a kind gift from Dr. Mark Hannink at the University of Missouri-Columbia, was transformed into *E. coli* BL21 (DE3) cells. The expression and purification were carried out using the procedure described in Khan et al. [77].

### 4.4. CD Spectropolarimetry

CD experiments were performed using a Jasco J-810 spectropolarimeter. Spectra of FL-Nrf2 (~0.3 mg/mL) and Neh1–2LZ1 construct (~0.1 mg/mL) were recorded in 50 mM ammonium acetate buffer, pH 6.5. FL-Nrf2 spectra were recorded at 5, 10, 25, and 35 °C. For each spectrum, 20 accumulated scans were obtained at 20 nm/min rate. For Neh1–2LZ1, the data were recorded at 20 °C with 20 accumulated scans (20 nm/min). The CD data were deconvoluted using the CONTINLL program in DichroWeb, with protein reference data set 4 (optimized for 190–240 nm) [78].

### 4.5. NMR Spectroscopy

^1^H-^15^N HSQC NMR spectra of FL-Nrf2 were acquired on Varian Inova 600-MHz spectrometers (UWO Biomolecular NMR Facility) at 5, 10, 25, and 35 °C in 50 mM ammonium acetate buffer (pH 6.5) using BioPack. Each data set was recorded with 160 scans and a relaxation delay of 1.0 s. For Neh1–2LZ1 (in ammonium acetate buffer, pH 6.5), spectra were recorded in the presence and absence of 50 mM arginine. Each spectrum was recorded with 32 scans, and a relaxation delay of 1.0 s. A total of 1 mM 4,4-dimethyl-4-silapentane-1-sulfonic acid (DSS) was added to the samples for chemical shift referencing. Data were processed and analyzed using NMRPipe and NMRViewJ, respectively [79,80].

### 4.6. HDX-MS

HDX-MS experiments were performed in 50 mM sodium phosphate buffer (90% D_2_O, pH 7.0) with 100 mM NaCl and at a final protein concentration of 2 μM for experiments on isolated FL-Nrf2 and Kelch. In Nrf2-Kelch-binding experiments, the FL-Nrf2 concentration was kept at 2 μM, and the Kelch concentration was varied from 2, 4, and 6 μM for 1:1, 1:2, and 1:3 (FL-Nrf2:Kelch) binding experiments. Aliquots were removed after 0.1, 0.2, 0.4, 5, 30, and 60 min and were quenched by lowering the pH to 2.5 using 20% (*v*/*v*) formic acid, followed by flash freezing in liquid nitrogen. The samples were thawed and injected into a nanoACQUITY UPLC equipped with HDX technology (Waters, Milford, MA, USA). Online digestion was carried out using a POROS pepsin column (2.1 × 30 mm, Life Technologies/Applied Biosystems) held at 15 °C. Peptic peptides were trapped on a C18 BEH130 VanGuard column (5 × 1 mm, 1.7 μm) for three minutes at 80 μL/min and separated on a C8 column (50 × 2.1 mm, 1.7 μm) at 100 μL/min using a water/acetonitrile gradient with 0.1% formic acid. The LC outflow was directed to a Waters Synapt Q-TOF G2 mass spectrometer. The ion source was operated at+2.8 kV and a cone voltage of 20 V. The desolvation and source temperatures were 250 and 80 °C, respectively. Mass spectra were acquired in resolution mode. Ion mobility was employed to aid in separating overlapping isobaric peaks.

Peptide identification was performed using three separate label-free MSE acquisitions with analysis using Protein Lynx Global Server 2.5.3. DynamX 3.0 was used for HDX data analysis. Deuterium uptake levels were corrected for artificial in-exchange and back-exchange using controls that represent minimum exchange under quench conditions (*m*_0_) and fully deuterated samples (*m*_100_), respectively. Percentage deuteration values for each peptide at time t was calculated according to

(1) $$\%D\left(t\right)=\frac{m_{t}-m_{0}}{m_{100}-m_{0}}\times 100\%$$

Pepsin digestion yielded 101 peptides for Nrf2, corresponding to 86.3% coverage. Digestion of Kelch yielded 109 peptides (99.7% coverage, see Figure S5B,D).

## Data Availability

The data presented in this study are available within the article text, figures, and Supplementary Materials.

## Supplementary Materials

The following are available online at https://www.mdpi.com/article/10.3390/ijms22147434/s1, Figure S1: SDS-PAGE analysis of the FL-Nrf2 at different purification steps, Figure S2: Bioinformatics predictions of the structural properties of FL-Nrf2, Figure S3: ^1^H-^15^N HSQC spectrum of FL-Nrf2 recorded at 35 °C in the presence of 6 M urea, Figure S4: The CD spectrum of Neh1–2LZ1 recorded at 20 °C, Figure S5: Peptide coverage of FL-Nrf2 and Kelch domain of Keap1, Figure S6: HDX data suggest the majority of FL-Nrf2 is not protected, Figure S7: The (A) SSD-AGE and (B) SDS-PAGE results of the sedimentation assay show that the FL-Nrf2 does not form aggregates at low concentrations, Table S1: Estimated secondary structural contents presence in FL-Nrf2 at different temperatures, The procedure of the sedimentation assay.
