# Supplementary material for: Nrf2, the Major Regulator of the Cellular Oxidative Stress Response, is Partially Disordered
Source: Int J Mol Sci. 2021 Jul 11;22(14):7434. doi: 10.3390/ijms22147434 (PMC8305528; doi:10.3390/ijms22147434)
Supplement: Supplementary file 1 [file ijms-22-07434-s001.zip › ijms-1285313-supplementary.pdf]

Supplemental Materials

Nrf2, the major regulator of cellular oxidative stress response, is partially disordered

Nadun C. Karunatilleke [1], Courtney S. Fast [2], Vy Ngo [3], Anne Brickenden [1],
Martin L. Duennwald [3], Lars Konermann [1,2,*] and Wing-Yiu Choy [1,2,*]

1 Department of Biochemistry, The University of Western Ontario, London, Ontario, Canada N6A 5C1
2 Department of Chemistry, The University of Western Ontario, London, Ontario, Canada N6A 5B7
3 Department of Pathology and Laboratory Medicine, The University of Western Ontario, London, Ontario, Canada N6A 5C1

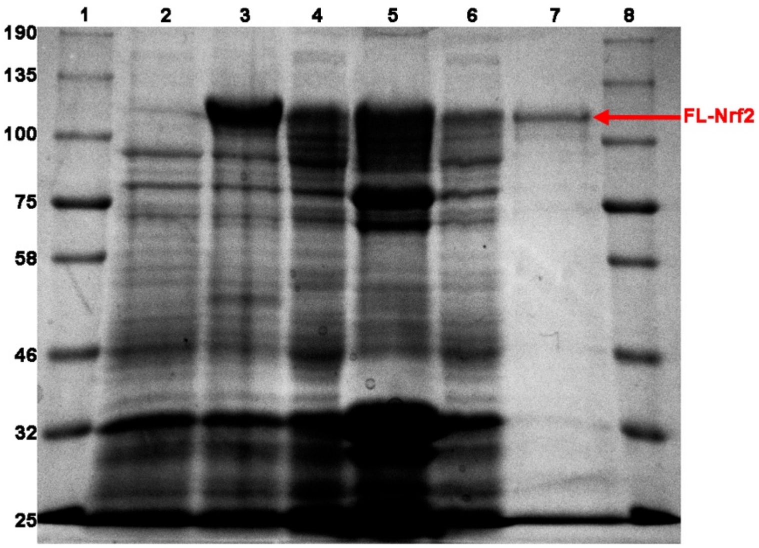

Figure S1. SDS-PAGE analysis of the FL-Nrf2 at different purification steps. Samples were run on a 10% polyacrylamide gel. Lane 1, molecular weight marker; lane 2, pre-induction sample; lane 3, post-induction sample; lane 4, soluble fraction; lane 5, insoluble fraction; lane 6, unbound fraction, lane 7, elution (pointed from red letters), and lane 8, molecular weight marker.

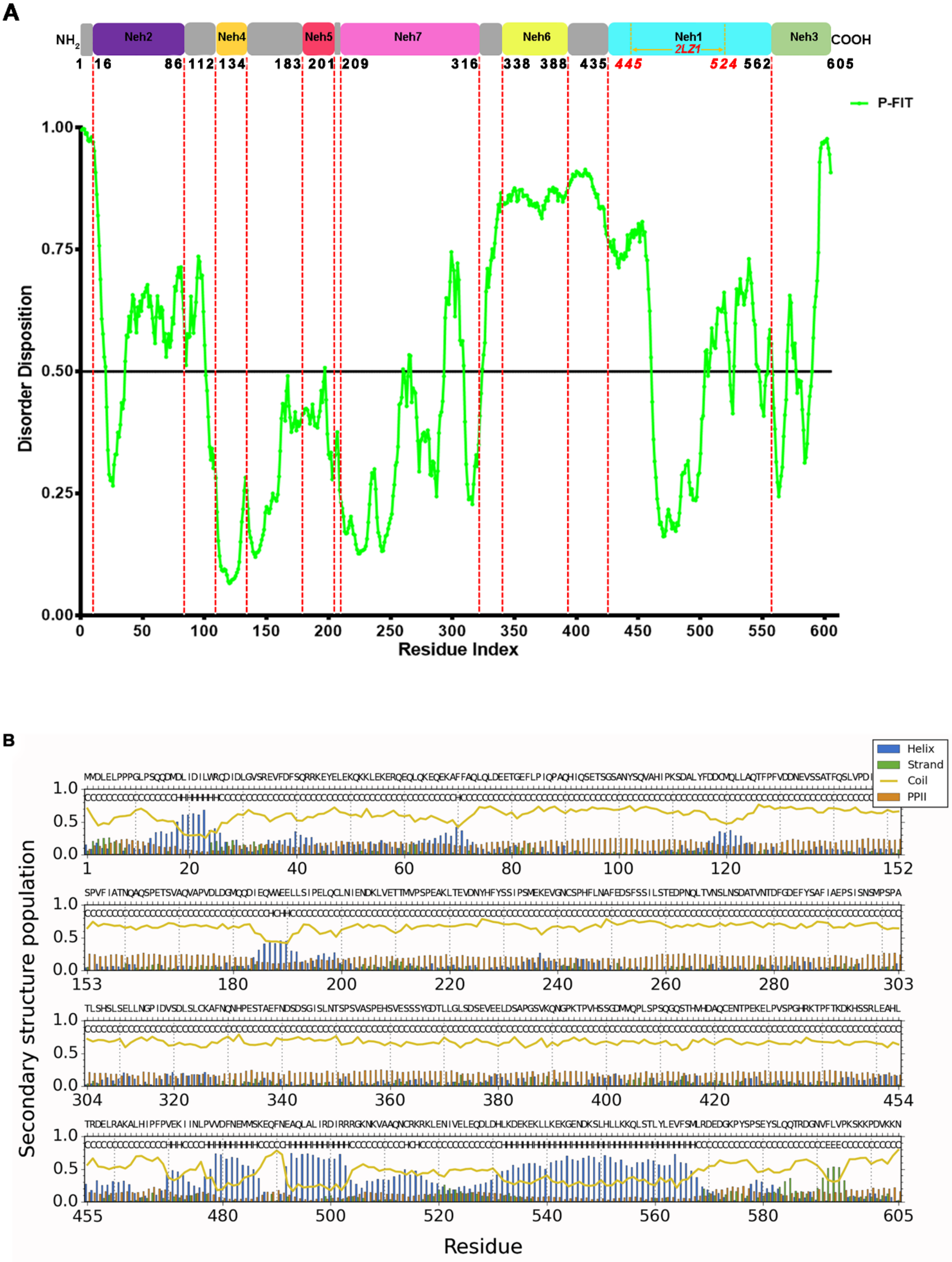
A
NH2
Neh2
Neh4
Neh5
Neh7
Neh6
Neh1
2LZ1
Neh3
COOH
1 16 86 112 134 183 201 209 316 338 388 435 445 524 562 605
P-FIT
Disorder Disposition
Residue Index
B
Helix
Strand
Coil
PPII
Secondary structure population
Residue

C

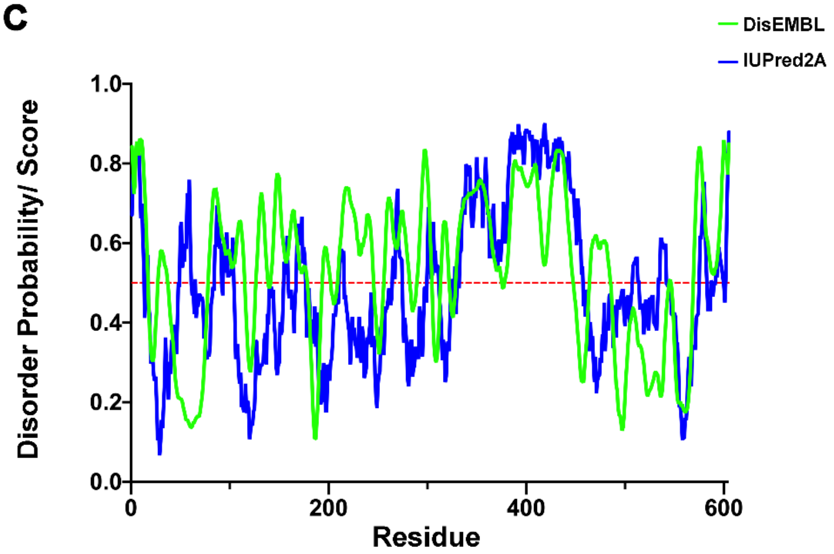

Figure S2. Bioinformatics predictions of the structural properties of FL-Nrf2. **(A)** Prediction of protein disorder with PONDR-FIT (P-FIT). Regions with y-axis values greater than 0.5 are predicted to be disordered. **(B)** Secondary structure propensity prediction of Nrf2 with s2D, **(C)** DisEMBL and IUPred2A disorder predictions.

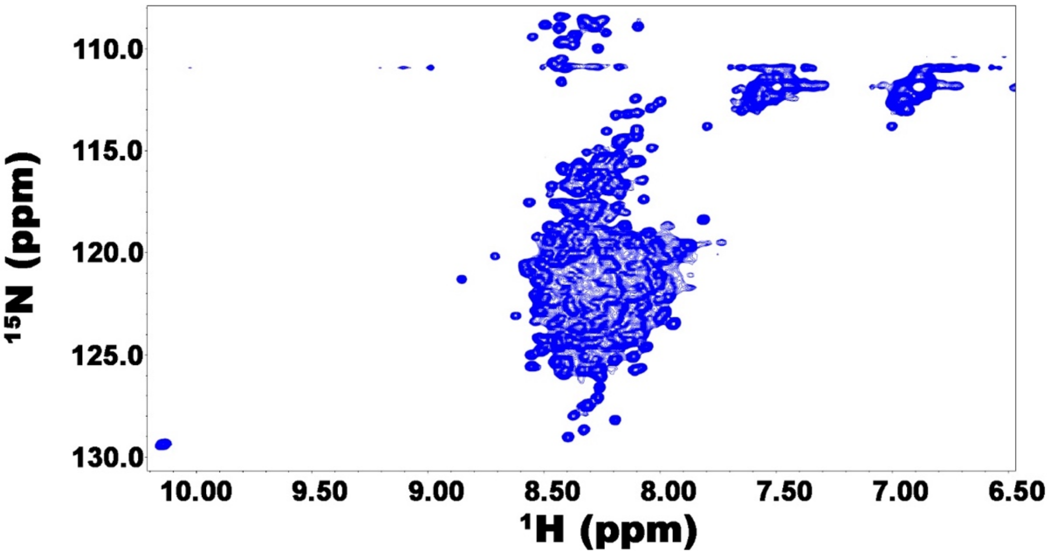

Figure S3. ^{1}H-^{15}N HSQC spectrum of FL-Nrf2 (~20 μM) recorded at 35 ºC in the presence of 6 M urea.

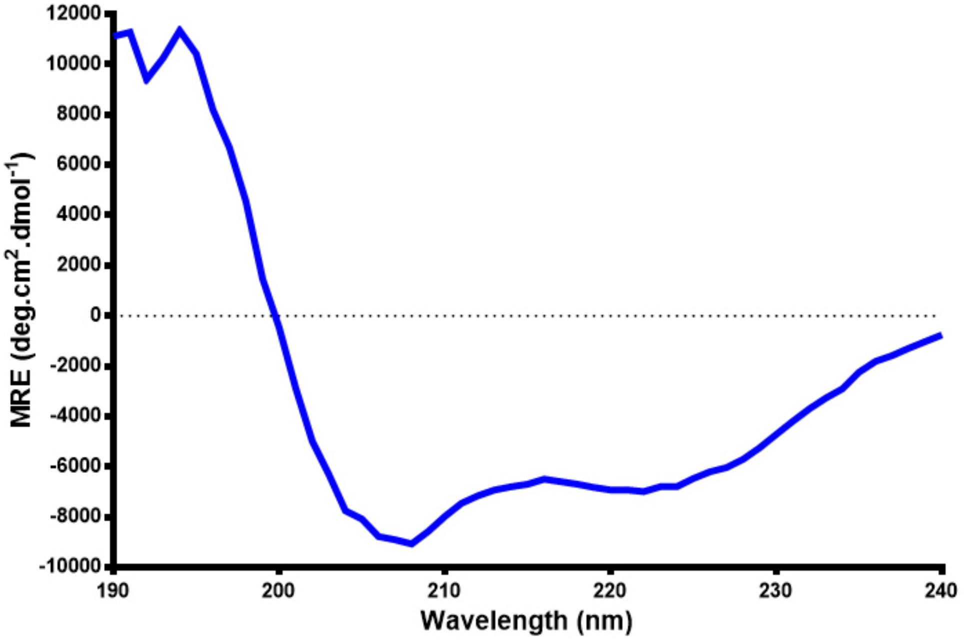

Figure S4. The CD spectrum (average of 20 scans) of Neh1-2LZ1 recorded at 20 ºC.

A

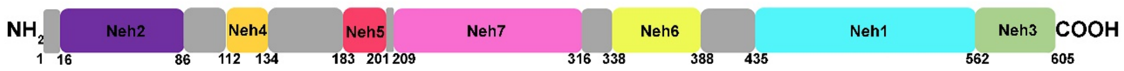

B

MS YYHHHHHHLE STSLYKKAGT

^{2}MDLELPPPG LPSQQDMDLI DILWRQDIDL GVSREVFDFS

QRRKEYELEK QKKLEKERQE QLQKEQEKAF FAQLQLDEET

GEFLPIQPAQ HIQSETSGSA NYSQVAHIPK SDALYFDDCM

QLLAQTFPFV DDNEVSSATF QSLVPDIPGH IESPVFIATN

QAQSPETSVA QVAPVDLDGM QQDIEQVWEE LLSIPELQCL

NIENDKLVET TMVPSPEAKL TEVDNYHFYS SIPSMEKEVG

NCSPHFLNAF EDSFSSILST EDPNQLTVNS LNSDATVNTD

FGDEFYSAFI AEPSOSMSMP SPATLSHSLS ELLNGPIDVS

DLSKCKAFNQ NHPESTAEFN DSDSGISLNT SPSVASPEHS

VESSSYGDTL LGLSDSEVEE LDSAPGSVKQ NGPKTPVHSS

GDMVQPLSPS QGQSTHVHDA QCENTPEKEL PVSPGHRKTP

FTKDKHSSRL EAHLTRDELR AKALHIPFPV EKIINLPVVD

FNEMMSKEQF NEAQLALIRD IRRRGKNKVA AQNCRKRKLE

NIVELEQDLD HLKDEKEKLL KEKGENDKSL HLLKKQLSTL

YLEVFSMLRD EDGKPYSPSE YSLQQTRDGN VFLVPKSKKP

DVKKN^{605}

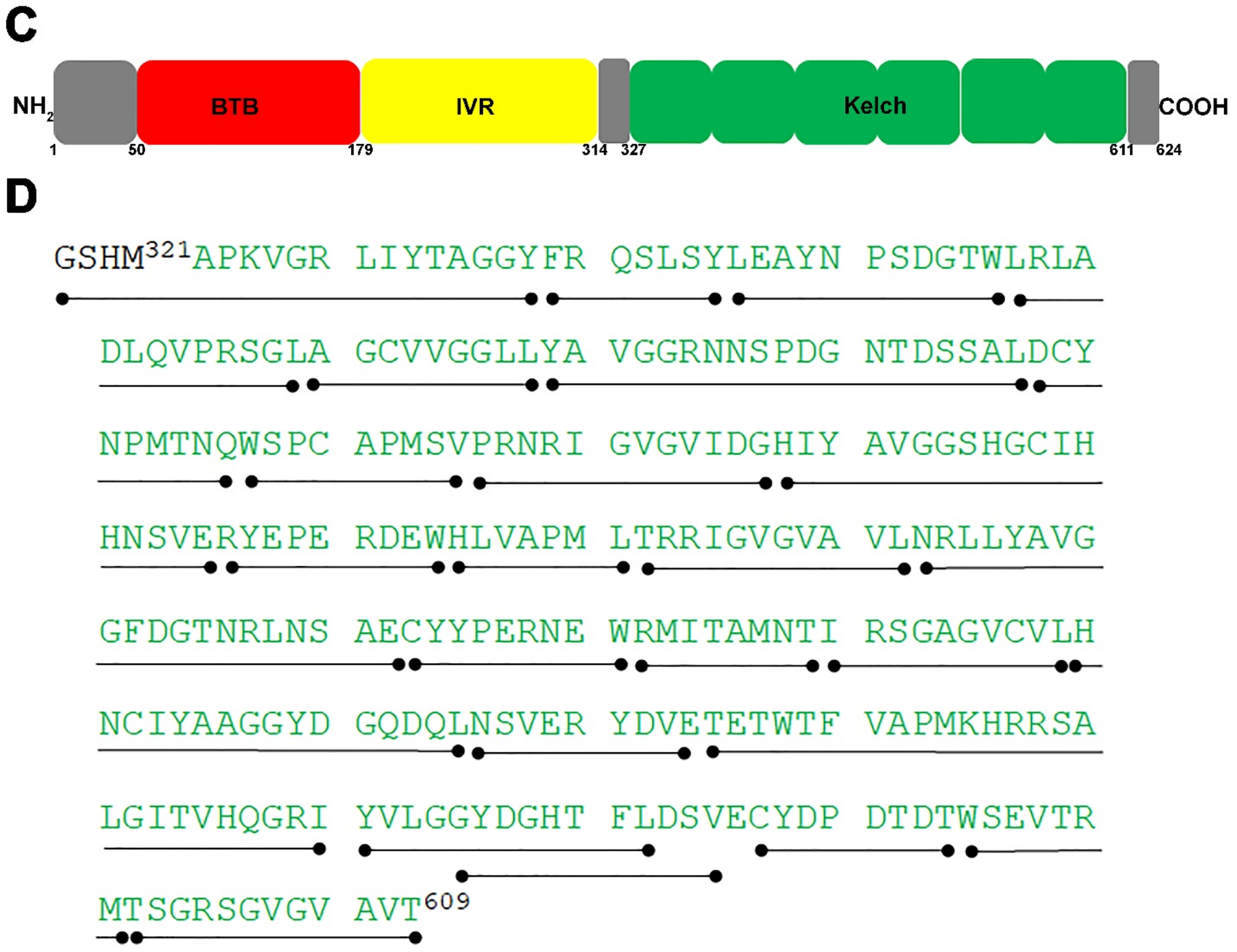

Figure S5. Peptide coverage of FL-Nrf2 and Kelch domain of Keap1. (A) FL-Nrf2 consists of seven domains: Nrf2-ECH homology (Neh) 1-7 domains **(B)** HDX-MS experiments of FL-Nrf2 yielded 101 peptides resulting in 86.3% of overall sequence coverage. MSYYHHHHHHLESTSLYKKAGT is the N-terminal His-tag **(C)** Keap1 consists of three domains: the BTB, IVR, and Kelch domain. **(D)** HDX-MS experiments of Kelch yielded 109 peptides resulting in 99.7% of overall sequence coverage. The N-terminal GSHM are residual residues from the removed His-tag.

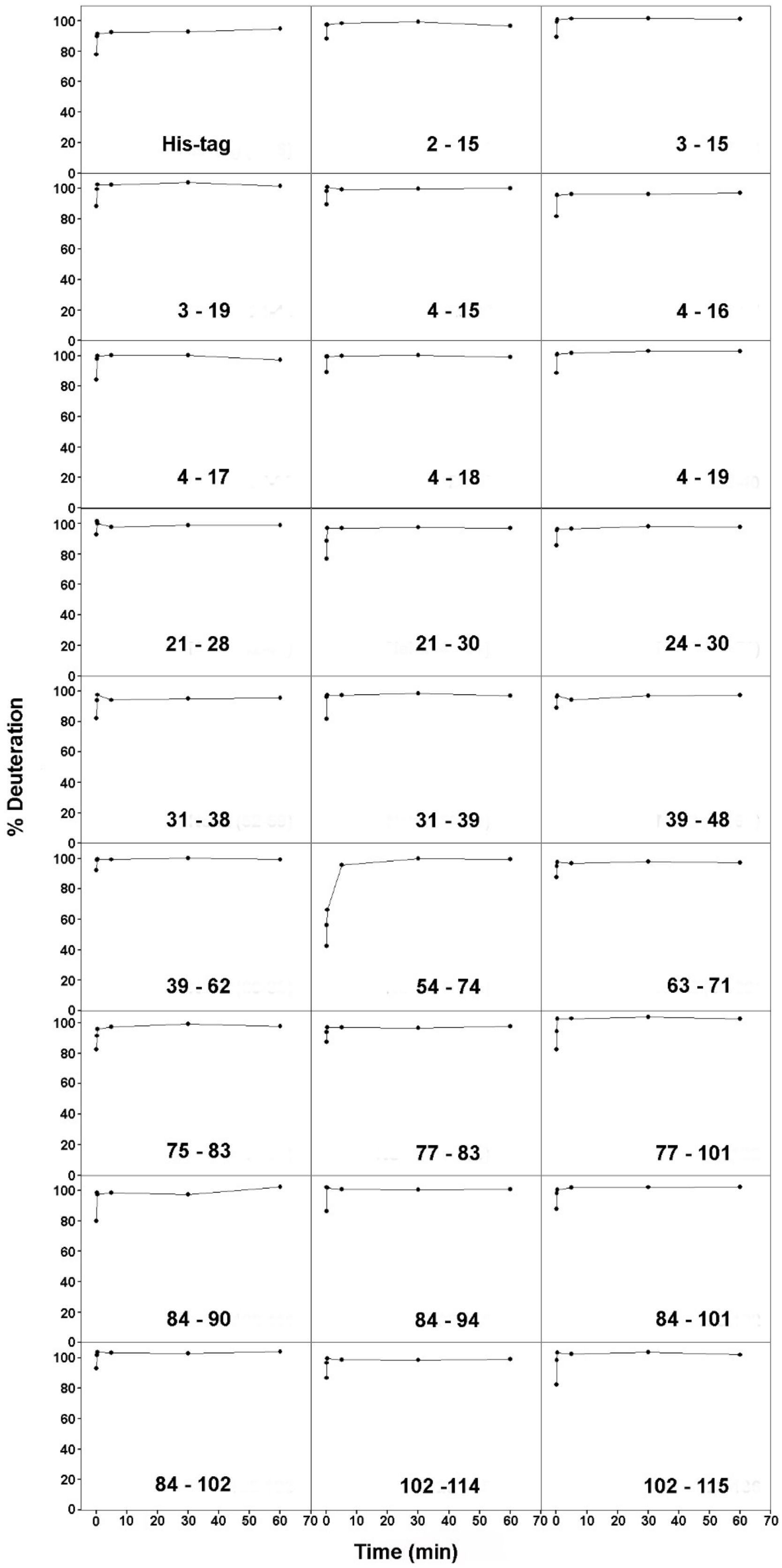

His-tag
2 - 15
3 - 15
3 - 19
4 - 15
4 - 16
4 - 17
4 - 18
4 - 19
21 - 28
21 - 30
24 - 30
31 - 38
31 - 39
39 - 48
39 - 62
54 - 74
63 - 71
75 - 83
77 - 83
77 - 101
84 - 90
84 - 94
84 - 101
84 - 102
102 -114
102 - 115
% Deuteration
Time (min)

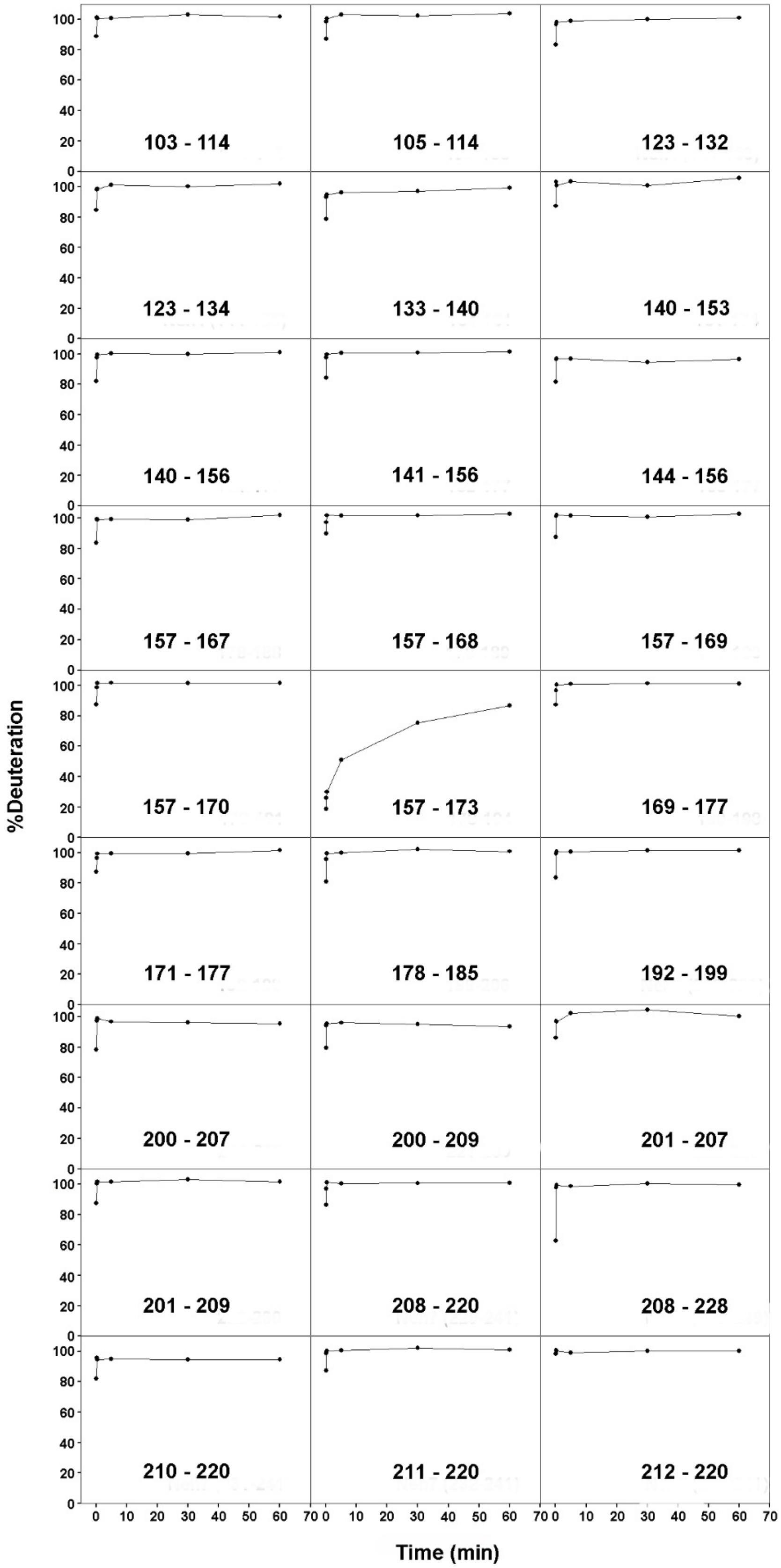
%Deuteration
Time (min)
103 - 114
105 - 114
123 - 132
123 - 134
133 - 140
140 - 153
140 - 156
141 - 156
144 - 156
157 - 167
157 - 168
157 - 169
157 - 170
157 - 173
169 - 177
171 - 177
178 - 185
192 - 199
200 - 207
200 - 209
201 - 207
201 - 209
208 - 220
208 - 228
210 - 220
211 - 220
212 - 220

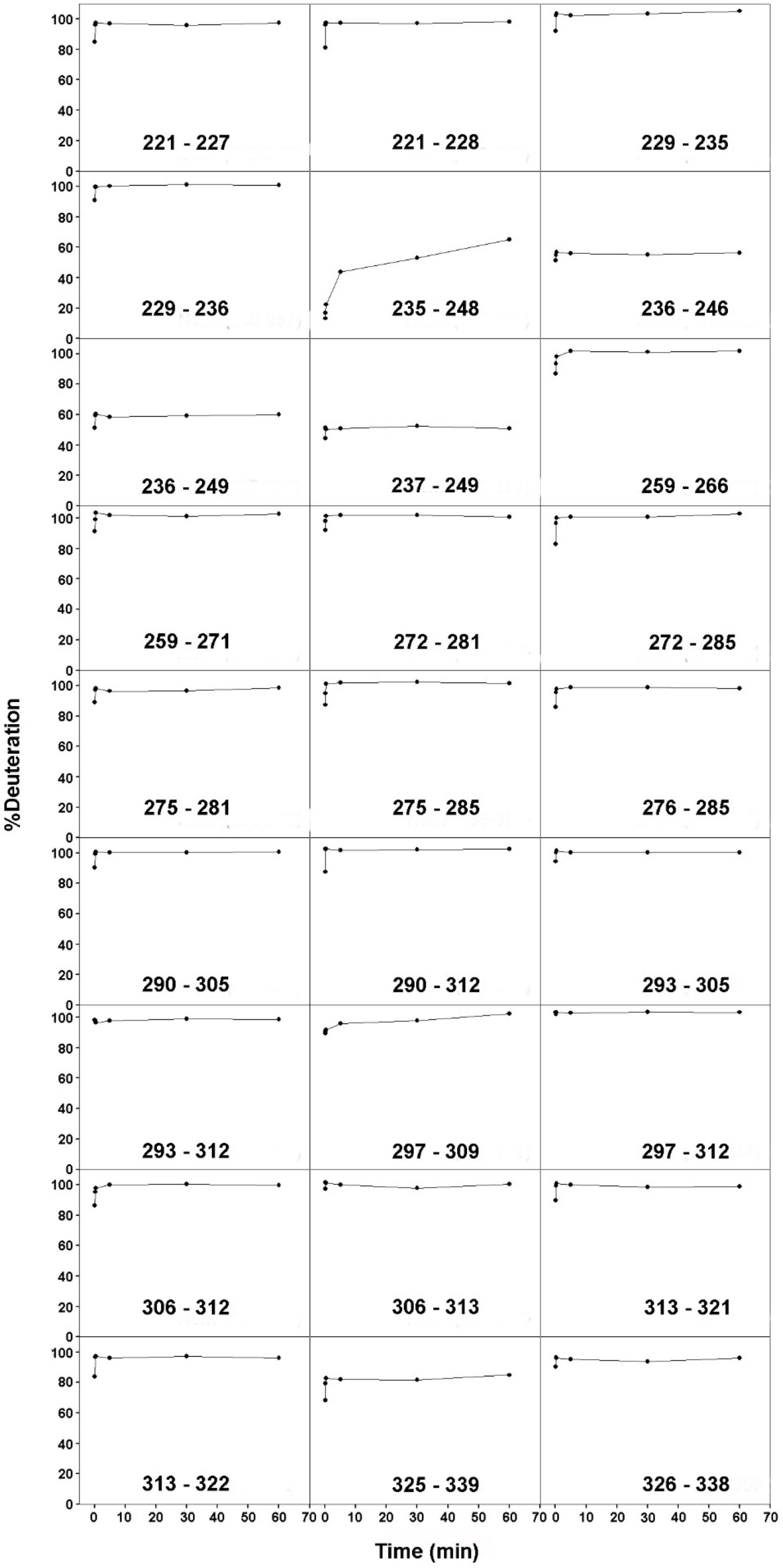
221 - 227
221 - 228
229 - 235
229 - 236
235 - 248
236 - 246
236 - 249
237 - 249
259 - 266
259 - 271
272 - 281
272 - 285
275 - 281
275 - 285
276 - 285
290 - 305
290 - 312
293 - 305
293 - 312
297 - 309
297 - 312
306 - 312
306 - 313
313 - 321
313 - 322
325 - 339
326 - 338
%Deuteration
Time (min)

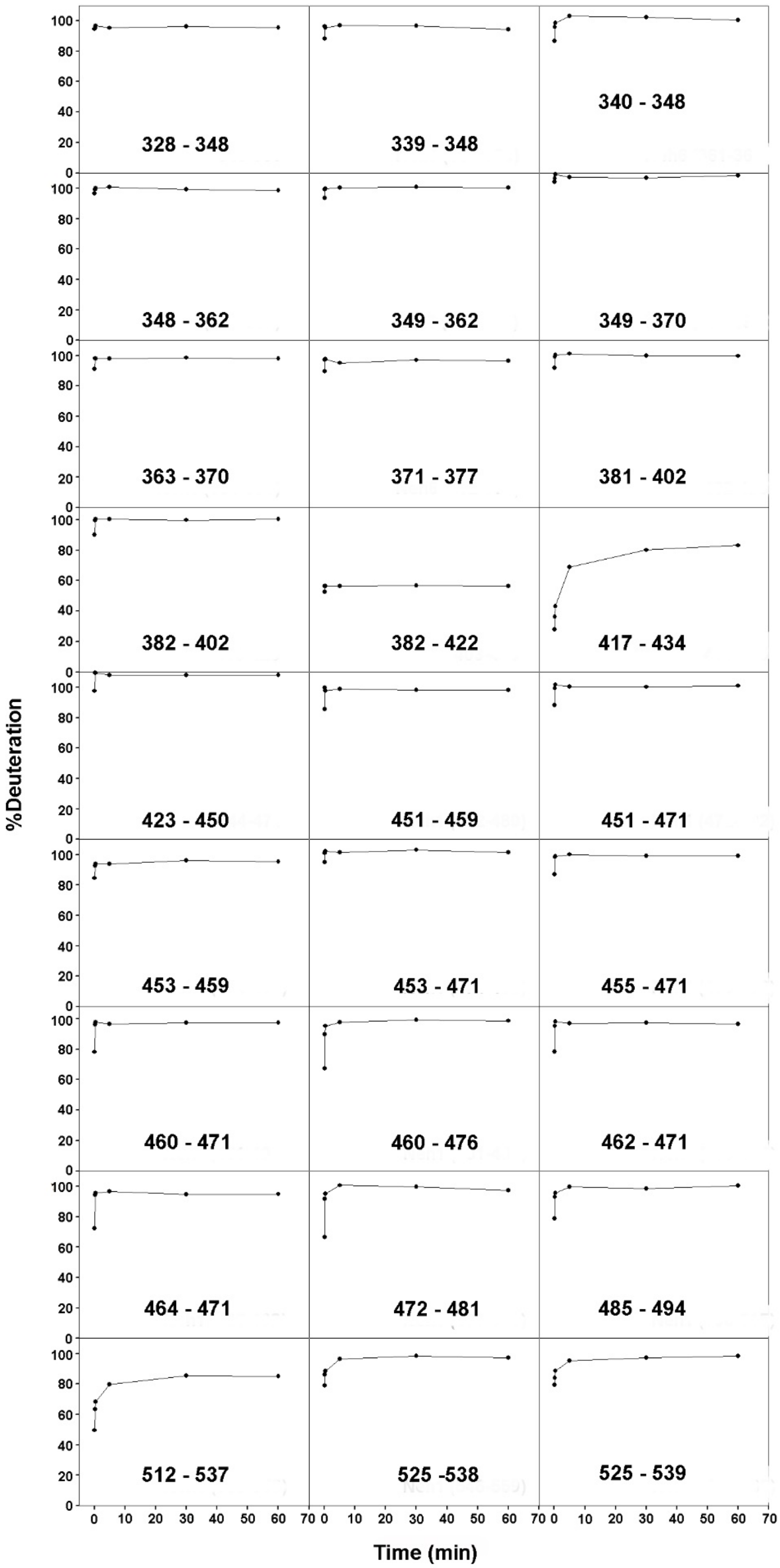

%Deuteration
Time (min)
328 - 348
339 - 348
340 - 348
348 - 362
349 - 362
349 - 370
363 - 370
371 - 377
381 - 402
382 - 402
382 - 422
417 - 434
423 - 450
451 - 459
451 - 471
453 - 459
453 - 471
455 - 471
460 - 471
460 - 476
462 - 471
464 - 471
472 - 481
485 - 494
512 - 537
525 -538
525 - 539

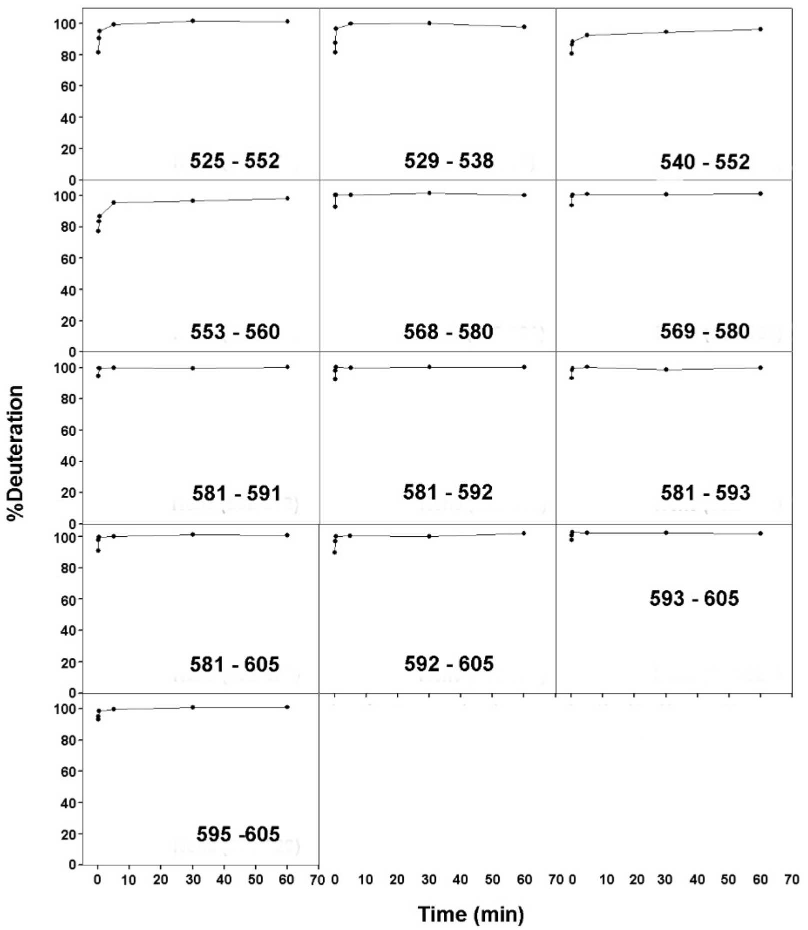

Figure S6. HDX data suggest the majority of FL-Nrf2 is not protected (Panels 1-5)

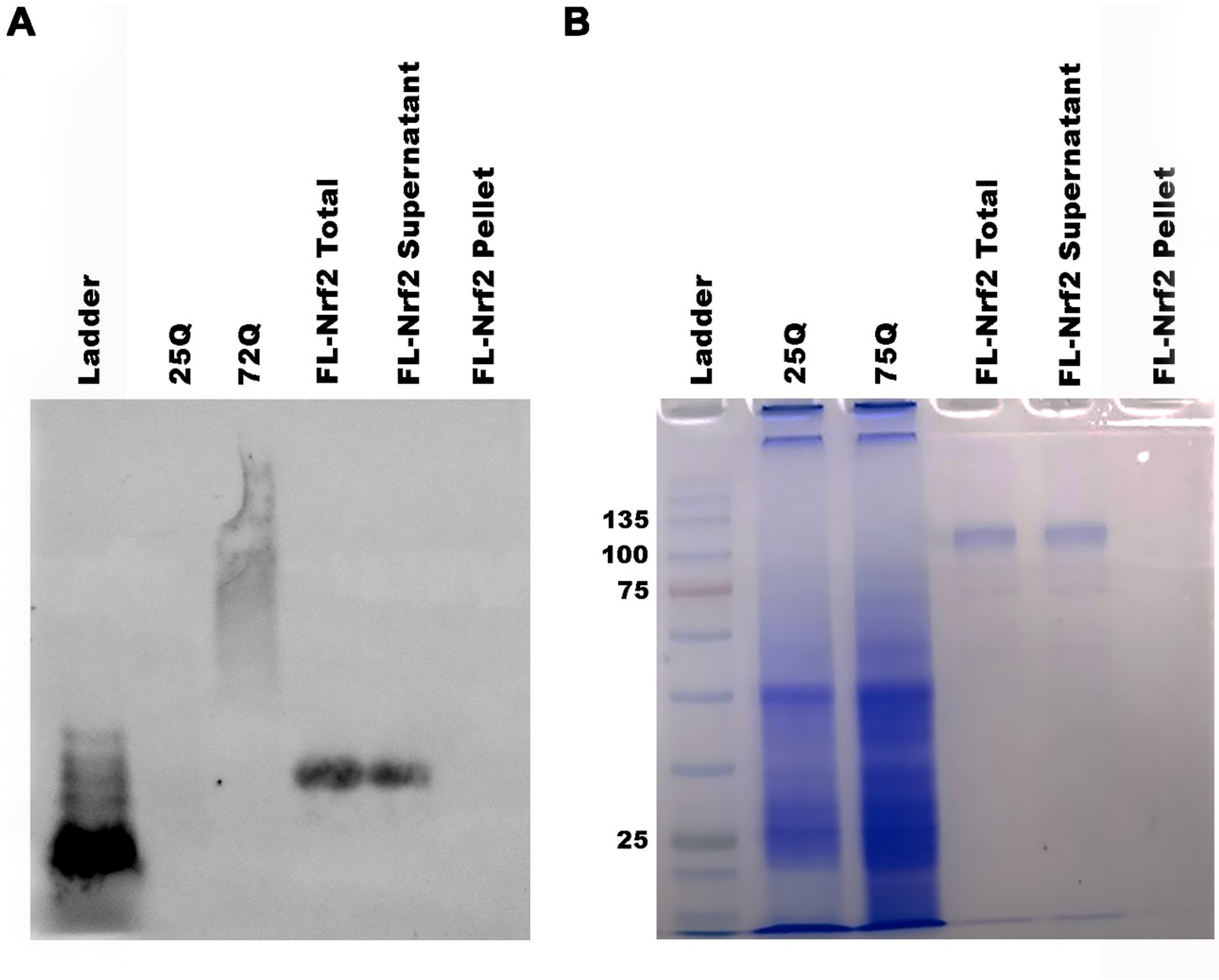

Figure S7. The **(A)** SSD-AGE and **(B)** SDS-PAGE results of the sedimentation assay show that the FL-Nrf2 does not form aggregates at low concentrations. The 25Q Htt protein, which does not aggregate, is used as the negative control, while the aggregated 75Q Htt is used as the positive control (The procedure of the sedimentation assay is outlined at the end of the Supplemental Materials).

Table S1. Estimate secondary structural contents presence in FL-Nrf2 at different temperatures. CD spectra were deconvoluted using DichroWeb. Analysis program CONTINLL with the reference set 4 was used for the deconvolution.

**Temperature (°C)**	**Estimated Secondary Structural Content (%)**		
	**Helix**	**Strand**	**Turns/Disordered**
5	28.9	16.8	54.3
10	30.0	15.5	54.5
25	26.7	21.0	52.3
35	17.7	26.9	55.4

The procedure of the sedimentation assay

The solubility of FL-Nrf2 was analyzed by the sedimentation assay to partition the soluble and aggregated protein molecules into supernatant and pellet for analysis.

Sample preparation

An aliquot of purified protein representing the "total" fraction was centrifuged at 10,000 x *g* for 10 minutes at 4 °C. The resultant supernatant represented the "supernatant" fraction; the pellet was resuspended in an equal volume of dialysis buffer and represented the "pellet" fraction. 2 µg of "total" purified protein and an equivalent volume of the "supernatant" and "pellet" fractions were boiled at 100 °C for 4 minutes and pulse-spun prior to SDD-AGE and SDS-PAGE analysis.

A positive control and a negative control were used along with the Fl-Nrf2 fractions. In Huntington's disease, abnormal expansion of the polyglutamine (polyQ) repeat in the huntingtin (Htt) protein confers toxic function(s) to mutant Htt, which can lead to neurodegeneration. The longer the polyQ expansion, the more toxic and aggregation-prone the Htt protein is. The 25Q Htt protein, which does not aggregate is used as the negative control, while the aggregated 75Q Htt is used as the positive control.

Semi-Denaturating Detergent Agarose Gel Electrophoresis **(***SDD-AGE)*

The protein samples were resolved on an 1.8% agarose-2% SDS gel. The transfer stacks were assembled according to the protocol provided by the supplier (Whatman TurboBlotter Transfer System), and the gel was transferred to PVDF by an overnight wet transfer by capillary force at room temperature with 1X TBS. The membrane was blocked with 5% milk in PBST and incubated with rabbit anti-Nrf2 (sc-722; Santa Cruz Biotechnology) primary antibody in 2% bovine serum albumin (BSA) overnight at 4 °C. The membrane was then incubated with goat anti-rabbit Alexa

Fluor 680 (A-21076; Invitrogen) secondary antibody for 1 h at room temperature. Images were taken using the Gel Doc XR+ System (Bio-Rad).

SDS-PAGE

Protein samples were resolved on a 10% SDS-PAGE gel. The gel was incubated in 50 mL of Coomassie Brilliant Blue staining solution (50% methanol (v/v), 10% glacial acetic acid (v/v), 40% H_2O, 1 g/L of Coomassie Brilliant Blue (Bio-Rad)) at room temperature for 30 minutes and destained with Coomassie Brilliant Blue destaining solution (40% methanol (v/v), 10% glacial acetic acid (v/v), 50% H_2O]) overnight.
